# Cortical lesions and focal white matter injury are associated with attentional performance in chronic traumatic brain injury

**DOI:** 10.1093/braincomms/fcae420

**Published:** 2024-11-21

**Authors:** Samuel B Snider, Natalie Gilmore, Holly J Freeman, Chiara Maffei, Alexander Atalay, Raj G Kumar, Lucia M Li, Hui Shi, Yelena G Bodien, Christine L Mac Donald, Kristen Dams-O’Connor, Brian L Edlow

**Affiliations:** Department of Neurology, Brigham and Women’s Hospital, Harvard Medical School, Boston, MA 02115, USA; Center for Neurotechnology and Neurorecovery, Department of Neurology, Massachusetts General Hospital, Harvard Medical School, Boston, MA 02114, USA; Center for Neurotechnology and Neurorecovery, Department of Neurology, Massachusetts General Hospital, Harvard Medical School, Boston, MA 02114, USA; Center for Neurotechnology and Neurorecovery, Department of Neurology, Massachusetts General Hospital, Harvard Medical School, Boston, MA 02114, USA; Athinoula A. Martinos Center for Biomedical Imaging, Department of Radiology, Massachusetts General Hospital, Harvard Medical School, Charlestown, MA 02129, USA; Center for Neurotechnology and Neurorecovery, Department of Neurology, Massachusetts General Hospital, Harvard Medical School, Boston, MA 02114, USA; Department of Rehabilitation and Human Performance, Icahn School of Medicine at Mount Sinai, New York, NY 10029, USA; Department of Brain Sciences, Imperial College London, W12 0BZ London, UK; Department of Neurology, Brigham and Women’s Hospital, Harvard Medical School, Boston, MA 02115, USA; Center for Neurotechnology and Neurorecovery, Department of Neurology, Massachusetts General Hospital, Harvard Medical School, Boston, MA 02114, USA; Department of Physical Medicine and Rehabilitation, Spaulding Rehabilitation Hospital, Harvard Medical School, Charlestown, MA 02129, USA; Department of Neurological Surgery, University of Washington, Seattle, WA 98195, USA; Department of Rehabilitation and Human Performance, Icahn School of Medicine at Mount Sinai, New York, NY 10029, USA; Department of Neurology, Icahn School of Medicine at Mount Sinai, New York, NY 10029, USA; Center for Neurotechnology and Neurorecovery, Department of Neurology, Massachusetts General Hospital, Harvard Medical School, Boston, MA 02114, USA; Athinoula A. Martinos Center for Biomedical Imaging, Department of Radiology, Massachusetts General Hospital, Harvard Medical School, Charlestown, MA 02129, USA

**Keywords:** attention, TBI, diffusion tensor MRI, lesions

## Abstract

Cognitive impairment, often due to attentional deficits, is a primary driver of disability after traumatic brain injury. It remains unclear whether attentional deficits are caused by injury to specific brain structures or the total burden of injury. In this cross-sectional, multicentre cohort study, we tested whether the association between brain injury and attentional performance varies by neuroanatomic location. Participants in the late effects of traumatic brain injury study were at least 18 years old and at least 1 year after a mild, moderate or severe traumatic brain injury. They underwent MRI and neuropsychological assessment at one of two sites. The primary and secondary outcomes, each measuring aspects of attentional performance, were the Trails A *t*-score and the standardized score on California Verbal Learning Test 2 Immediate Recall Trial 1. Imaging variables included the size and location (seven regions and seven networks) of encephalomalacic brain lesions and regional white matter fractional anisotropy measured with diffusion MRI (14 regions). We used ANOVA to test whether attentional performance differed by lesion location and linear mixed models to test whether attentional performance differed based on regional fractional anisotropy. One hundred eighty-eight participants met inclusion criteria (mean age 57, 69% male, 88% White). Participants with encephalomalacic brain lesions [*N* = 73 (39%)] had worse Trails A [mean (95% confidence interval) difference: 4.7 (0.3, 9.1); *P* = 0.036] but not secondary outcome performance [−0.3 (−0.1, 0.7); *P* = 0.17]. Among participants with lesions, Trails A performance did not differ by lesion size (*P* = 0.07) or location (*P* = 0.41 by region; *P* = 0.78 by network). We identified a significant interaction between regional fractional anisotropy and attentional performance on both primary (*P* = 0.001) and secondary (*P* = 0.001) outcome measures. *Post hoc* testing identified the strongest associations with Trails A performance in the sagittal stratum [1 SD decrement in Trails A: −0.2 (−0.3, −0.1) SD change in fractional anisotropy; *P*_Bonferroni_ = 0.0057] and external capsule [−0.1 (−0.2, −0.1); *P*_Bonferroni_ = 0.042] and the strongest association with secondary attentional scores in the corpus callosum [0.2 (0.1, 0.3); *P*_Bonferroni_ = 0.014]. In a multivariate model, white matter integrity in the sagittal stratum (*P* = 0.008), but not encephalomalacic lesions (*P* = 0.3), was independently associated with Trails A performance. Diminished white matter integrity and cortical injury were each associated with attentional test performance, but only white matter injury demonstrated independent and region-specific effects. The peak statistical association with attentional test performance was in the sagittal stratum, a widely connected white matter region. Further investigation into the connections spanning this and nearby regions may reveal therapeutic targets for neuromodulation.

## Teaser

Snider *et al*. report that cortical injury and diminished white matter integrity were each associated with attentional impairment in patients with chronic traumatic brain injury. Region-specific effects were only observed in the white matter. The strongest association with attentional impairment was identified in the sagittal stratum, a widely connected region.

## Graphical Abstract

**Graphical Abstract:**
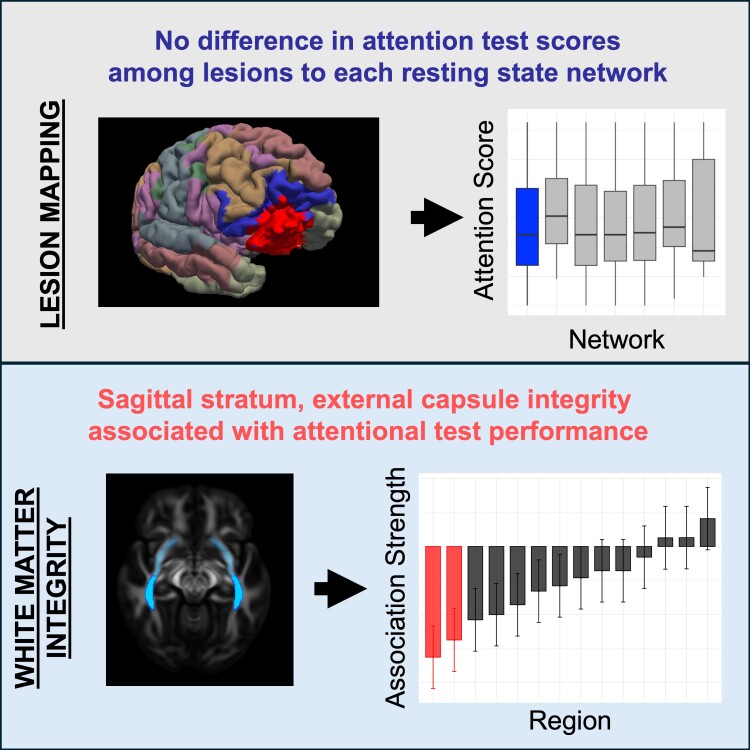

## Introduction

Cognitive impairment is a primary contributor to an individual’s level of dependency after traumatic brain injury (TBI).^1-3^ Attention is foundational to multiple cognitive processes,^4,5^ and attentional deficits are common in individuals with post-TBI cognitive impairment.^2,6^ While attentional performance may be influenced by psychological factors (post-traumatic stress disorder, depression)^7^ not directly related to focal brain injury,^8,9^ identifying a specific neuroanatomic signature of post-TBI attentional impairment would improve the accuracy of clinical prognostication and provide treatment targets to promote functional recovery in people with cognitive impairment after TBI.

After a TBI, a key unanswered question is whether attentional impairment results from the global burden of cerebral injury or focal damage to specific structures or networks. The mesocircuit hypothesis^10^ posits that reciprocal connections between the central thalamus, striatum and frontoparietal networks are important for sustaining arousal and attention. Several studies have identified connections and activity patterns in this circuit that suggest a role in sustained attention.^11-14^ However, empirical studies in people living with attentional impairment after TBI either do not implicate specific regions^15^ or point to other networks.^16-19^ Whether or not post-TBI attentional impairment has a specific underlying neuroanatomic signature has therapeutic implications. If attentional impairment after TBI results from injury to a specific node or network, then targeted neuromodulatory therapies may be beneficial.^20^ On the other hand, if attentional impairment after TBI results from the total burden of injury, then pharmacologic interventions that modulate distributed neurotransmitter systems may be efficacious.^21^

The neuropathology of chronic TBI includes encephalomalacia (confluent areas of necrotic tissue at the site of haemorrhagic contusions) and disrupted white matter integrity (resulting from axonal injury).^22-24^ Encephalomalacia can be identified on T1-weighted MRI sequences,^22,25^ and disrupted white matter integrity can be identified as reduced fractional anisotropy (FA) measured with diffusion MRI (dMRI).^22,24^ Here, we investigate the neuroanatomic correlates of attentional test performance in the late effects of TBI (LETBI) study, a prospective, multicentre, longitudinal study of individuals with chronic TBI in the USA.^26^ In this cohort, we sought to determine whether attentional test performance is associated with a specific neuroanatomic pattern of injury. Analysing both cortical encephalomalacia and subcortical white matter integrity, we test whether associations between structural injury and attentional performance vary by location within the brain.

## Materials and methods

### Cohort characteristics

We conducted a cross-sectional cohort study of participants enrolled in the LETBI study at two academic medical centres (Mount Sinai, New York, NY, USA and University of Washington, Seattle, WA, USA) between 2014 and 2022. Study procedures have been described previously.^26^ Briefly, eligible participants were at least 18 years old and at least 1 year after a complicated mild, moderate or severe TBI.^27^ These criteria were operationally defined as self-report or medical record evidence of a head trauma that resulted in at least one of the following: (i) Glasgow Coma Scale (GCS) score < 13 on emergency department arrival, loss of consciousness lasting at least 30 min, post-traumatic amnesia lasting over 24 h or any trauma-related intracranial abnormality on neuroimaging or (ii) a history of two or more head traumas resulting in loss of consciousness < 30 min, post-traumatic amnesia < 24 h and GCS score ≥ 13 on emergency department GCS score.^26^ The rationale for including individuals with multiple mild TBIs, as opposed to a single mild TBI, is based on histopathological studies that describe complex neurodegenerative pathologies in individuals exposed to single moderate-to-severe TBI or repetitive mild TBI, including chronic traumatic encephalopathy. While there is ongoing debate about whether a single mild TBI predisposes individuals to neurodegenerative conditions, we decided at the time of LETBI study initiation to focus on TBI-related exposures that are more tightly linked to neurodegenerative pathology based upon current evidence.

Among 301 patients enrolled through 2022, our analytic sample included *N* = 188 participants (missing MRI: 83 and missing Trail Making Test: 30; Fig. 1) with a T1-weighted MRI sequence and the primary behavioural measure completed (lesion analysis cohort) and *N* = 182 with a dMRI and completed primary behavioural measures (dMRI analysis cohort). The LETBI study was approved by Institutional Review Boards at each site, and participants or their legal representatives provided informed consent.

**Figure 1:**
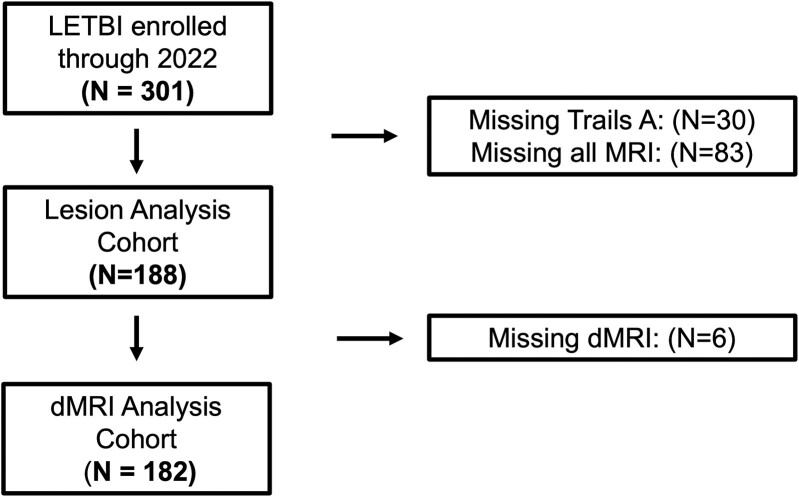
**Study flowchart.** Study CONSORT diagram. dMRI, diffusion MRI; LETBI, Late Effects of Traumatic Brain Injury.

### Primary outcome measure

As described previously,^26^ participants underwent assessment with a standard set of neuropsychological measures at the initial study visit. The focus of this study was attention, which underlies many cognitive processes and is difficult to measure in isolation.^28,29^ The primary outcome was attentional performance, as measured by time to completion of the Trails A^30^*t*-score (normalized to age and level of education).^31^ Trails A test is a commonly used cognitive outcome measure in studies of cognition after TBI^17,32-34^ and was recently used as an outcome measure in a Phase 1 study of thalamic deep brain stimulation for cognitive impairment.^35^ However, in addition to visuospatial attention, Trails A performance also depends on processing speed, motor dexterity and executive function, which may also be impaired by TBI.^32,36^ The secondary outcome measure, probing a different dimension of attention, was the California Verbal Learning Test^37^ 2 (CVLT) Immediate Recall Trial 1 standardized score (i.e. repeat 16 words back in any order immediately following auditory presentation; a measure of simple auditory attention).^38^ The CVLT Trial 1 is also an impure measure of auditory attention, with fundamental dependencies on language and short-term memory.^39^

### Imaging analysis

#### Traumatic brain lesions

Participants were scanned on either a Siemens Skyra 3T, Philips Achieva 3T or Philips Ingenia Elition 3T. Encephalomalacic brain lesions due to TBI were manually traced on high-resolution T1 multi-echo magnetization-prepared rapid acquisition gradient echo^40^ sequence. Two expert raters trained in neuroanatomy and imaging segmentation (N.G. and S.B.S.) initially screened all T1-weighted images for the presence of lesions. Encephalomalaic lesions were defined as contiguous areas of disrupted cortex and white matter that did not conform to a vascular distribution. We did not consider lesions restricted to the white matter or basal ganglia, which often have a microvascular provenance. We did, however, include the full extent of the lesion, including any deeper involvement (cingulate or basal ganglia), in the lesion label.

The lesions were subsequently traced manually by a programmer trained in imaging segmentation (H.J.F.) and checked for accuracy by N.G. We registered Montreal Neurologic Institute (MNI) atlas^41^ regions of interest (ROIs), thresholded at 30% and binarized from MNI152 T1 1 mm space onto participant T1 volumes using linear (rigid + affine) and non-linear (deformable syn) registrations (ANTs), and computed the volumetric overlap. In the same manner, we registered the seven canonical resting-state functional networks from the Yeo atlas^42^ onto the participant T1 volumes and computed the volumetric overlap.

#### dMRI acquisition and pre-processing

Single-shell dMRI acquisition parameters differed slightly, as detailed in Supplementary Table 1. All scans included two acquisitions with reverse phase encoding directions. The number of diffusion-encoding directions ranged from 30 to 64, the *b*-value was 1000 s/mm^2^ for all scans, and the spatial resolution was 2 mm isotropic for two scanners and 1.875 × 1.875 × 2 mm on the third (Supplementary Table 1).

Our pre-processing pipeline included denoising and deringing (dwidenoise,^43^ MRITRIX), top-up (fMRI Software Library) for susceptibility-induced distortions and eddy (fMRI Software Library) for eddy current effects and motion correction. Motion parameters and contrast-to-noise ratio were estimated using eddy (fMRI Software Library). Diffusion parameters were estimated using DTIFIT (fMRI Software Library). We used linear (rigid + affine) and non-linear (deformable syn) registrations from each participant’s FA map to the T1 multi-echo magnetization-prepared rapid acquisition gradient echo volume to the MNI 152 T1 1 mm space and finally to the Human Connectome Project 1065 subject mean FA map^44^ in MNI 1 mm space (ANTs; antsRegistrationSynQuick^45^). We visually checked each registration to confirm anatomic fidelity. In four participants, we achieved better alignment by performing a single registration from the participant’s FA map directly to the Human Connectome Project 1065 FA MNI map and used this direct registration for all subsequent analyses.

#### dMRI: white matter integrity

We used the inverse of the above transformations to map standard white matter ROIs from MNI space onto participant FA maps. White matter ROIs included 14 tract labels from the Johns Hopkins University ICBM-DTI atlas.^46^ To minimize degrees of freedom in subsequent analyses, we combined bilateral ROIs into a single label, collapsed all subsets of the cingulum, corona radiata and callosum and combined the middle and superior cerebellar peduncle labels and the posterior limb of the internal capsule with the retrolenticular limb of the internal capsule. We performed no manual editing of registered ROIs. We excluded the fornix due to the inability to reliably register this white matter tract accurately onto participant FA maps.

To measure white matter integrity, we thresholded the participant FA map at 0.2, as voxels below this level are unlikely to represent white matter.^47^ The measure of white matter integrity within each ROI was then defined as the mean FA of all remaining voxels within the ROI. Finally, we scaled each ROI’s FA distribution to unit variance (Supplementary Fig. 1) and excluded individual measurements > 3 SDs from the mean, as such outliers may reflect misregistered ROIs.

There are many well-documented^48,49^ sources of variance in measured FA that are unrelated to traumatic white matter injury. As described above, MRIs included in this study came from three different scanners, each with a slightly different acquisition protocol (Supplementary Table 1). To assess the degree to which variables unrelated to brain trauma may have influenced the whole white matter mean FA signal, we fit a linear model with a set a of candidate variables. These included age, scanner type (Skyra, Acheiva or Ingenia), contrast-to-noise ratio of the b0 volume, mean absolute motion between diffusion volumes and percentage of outlier slices (Supplementary Table 2). We then controlled for any independently significant (Supplementary Table 2; bolded entries) variables in the dMRI analyses described below.

### Statistical analysis

#### Association between attention and encephalomalacic lesions

All statistical analyses were performed in R (RStudio 1.4.1717). To determine if brain lesions affect attentional performance, we compared Trails A *t*-scores in patients with or without brain lesions using a two-sample *t*-test. In participants with brain lesions, we grouped lesion volumes by quartile and used a one-way ANOVA to determine whether attentional performance differed across volume groups. Required assumptions including residual normality (Supplementary Fig. 2) and homogeneity of variance (Levene’s test) were checked and confirmed. To determine whether there was an association between attentional performance and lesions to specific MNI atlas locations or resting-state functional networks, we compared Trails A *t*-score across participants with lesions to each structure/network using one-way ANOVA. ANOVA assumptions were again confirmed (Supplementary Fig. 3).

#### Association between attention and white matter integrity

To test whether associations between white matter integrity (scaled FA) and attention differed by white matter region, we used a linear mixed-effects model (R: lme4). This approach was well suited to the nested structure of our data (14 ROIs per subject). The random effect was subject, and fixed effects included nuisance variables [age, scanner type (Skyra, Acheiva or Ingenia), contrast-to-noise ratio of the b0 volume and mean absolute motion between diffusion volumes), as well as the main effects of interest: attentional test performance (Trails A *t*-score or CVLT Trial 1 standardized score), ROI and their interaction.

To test whether the association between attentional test performance and white matter integrity varied by region, we applied a mixed ANOVA to the model to evaluate the statistical significance of the attentional score × ROI interaction term. If the interaction term was found to be significant (*P* < 0.05), we then conducted *post hoc* tests across all ROIs, comparing estimated marginal associations between attentional performance and scaled FA, while adjusting for the other fixed and random effects (R: emtrends). We used a Bonferroni adjustment to correct for multiple comparisons during *post hoc* testing (14 ROIs).

To ensure that the regional associations with the primary outcome were not confounded by motor performance or time between injury and cognitive assessment, we conducted a series of sensitivity analyses adding covariates to the primary linear mixed-effects model. These included generalized physical functioning [Physical Functioning *t*-score from the Rand 36 Item Short Form Survey (SF-36)^50^], the presence of physical limitations [Physical Limitations *t*-score (SF-36)], motor parkinsonism (Part III of the Unified Parkinson’s Disease Rating Scale^51^*t*-score), dominant hand grip strength (*t*-score)^52^ and time between study visit and brain injury. Finally, to rule out hemisphere-specific associations, we fit a model that included all non-midline ROIs split by hemisphere. We tested for an interaction between the primary outcome × hemisphere and between primary outcome × ROI × hemisphere.

#### Encephalomalacic lesions versus white matter integrity

Finally, to address whether the presence of encephalomalacic lesions or white matter integrity was independently associated with attentional test performance, we fit a generalized linear model to the primary outcome (Trails A *t*-score), with predictors including age, scanner, contrast-to-noise ratio, motion, lesion (present/absent) and mean FA within the ROI with the most-significant marginal association from the previous analysis.

## Results

### Study cohort and cognitive outcomes

The final analysis cohort included *N* = 188 LETBI participants with chronic TBI who completed the cognitive assessment and underwent T1-weighted MRI (lesion analysis cohort) and *N* = 182 who additionally completed dMRI (dMRI analysis cohort; Fig. 1). For simplicity, we report the baseline characteristics of the lesion analysis cohort here, but the characteristics of each of these nearly identical cohorts are provided in Table 1. The mean age was 57 ± 15 years old, 69% of participants were male, and 89% were White (Table 1). The mean age- and education-standardized attentional performance (Trails A *t*-score) was 54 ± 15 (Table 1). Compared with participants with missing data, subjects with complete data were slightly more likely to be male (69% versus 55%; *P* = 0.02) and less likely to have attended college (68% versus 80%; *P* = 0.03).

**Table 1:** Cohort characteristics

		LETBI study (*N* = 301)	dMRI analysis cohort (*N* = 182)	Lesion analysis cohort (*N* = 188)	Missing behaviour/MRI (*N* = 113)	Difference *P*-value^a^
Mean age (SD)	57 (16)	57 (15)	57 (15)	57 (18)	0.8
Male sex (%)	189 (63)	124 (68)	129 (69)	60 (55)	**0**.**02**
White race (%)	258 (87)	161 (89)	166 (88)	92 (84)	0.3
Attended college (%)	214 (72)	122 (68)	126 (68)	88 (80)	**0**.**03**
Marital status (%)					0.6
Never married	75 (25)	44 (24)	45 (24)	31 (28)	
Married/partnered	130 (43)	78 (43)	81 (44)	49 (45)	
Divorced/widowed	91 (30)	58 (32)	60 (32)	31 (28)	
Employment (%)					0.8
Working/student	96 (32)	60 (33)	62 (33)	34 (31)	
Unemployed	18 (6)	10 (6)	10 (5)	8 (7)	
Retired	88 (30)	52 (29)	55 (30)	33 (30)	
Disabled	74 (25)	44 (24)	45 (24)	29 (26)	
Other	20 (7)	14 (8)	14 (8)	6 (5)	
Median years since most recent TBI (IQR)	8 (3, 18)	8 (3, 18)	8 (3, 17)	8 (4, 19)	0.5
Mean Trails A *t*-score (SD)	55 (15)	54 (14)	54 (15)	57 (17)	0.09
Impaired attention (%)^b^	67/271 (25)	43/182 (24)	46/188 (24)	21/83 (25)	0.7
Mean CVLT Immediate Recall Trial 1 Std. Score (SD)	−0.5 (1.3)	−0.5 (1.3)	−0.5 (1.3)	−0.6 (1.2)	0.7
Missing					
Behaviour (%)	30 (10)	26		30 (27)		
Imaging (%)	89 (30)	90	6 (3)	83 (73)		

Bold denotes *P*-value < 0.05.

CVLT, California Verbal Learning Test; dMRI, diffusion MRI; LETBI, late effects of TBI; SD, standard deviation; Std, standardized; TBI, traumatic brain injury.

^a^Each analysis cohort separately test against missing cohort; lowest *P*-value reported here.

^b^>1.5 SD above population mean (>65).

### Traumatic brain lesions and attentional test performance

**Figure 2:**
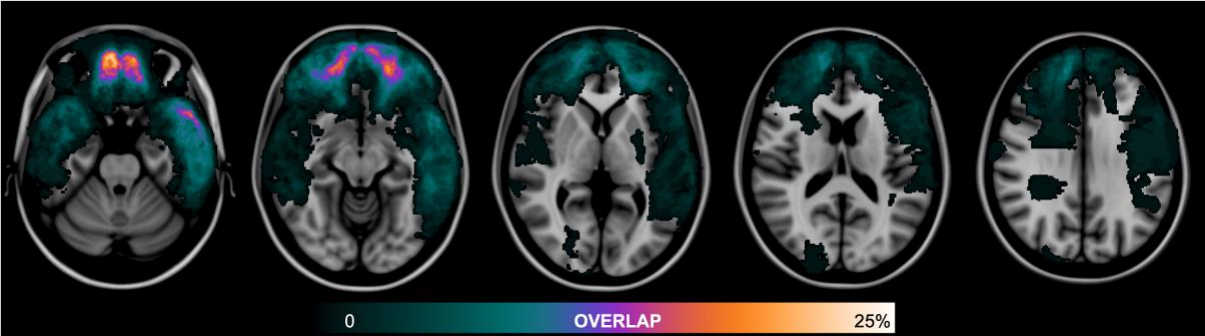
**Cortical lesion overlap map.** Encephalomalacic lesions are overlaid on an MNI template brain. The colour bar indicates the percentage of the cohort with a lesion at each voxel.

Seventy-three participants (39%) had chronic encephalomalacic lesions on T1-weighted MRI. Lesions were distributed primarily throughout the inferior frontal and temporal lobes (Fig. 2). Compared with those without lesions, participants with lesions had worse attentional performance [Trails A *t*-score mean difference: 4.7, 95% confidence interval: (0.3, 9.1); *P* = 0.036; Supplementary Fig. 4). Among participants with brain lesions, there was no difference in Trails A performance between lesion volume quartile (*F*(3,70) = 2.4; *P* = 0.07; Supplementary Fig. 5). Furthermore, Trails A performance did not differ between participants with lesions of each cortical lobe or the basal ganglia (*F*(6,432) = 1.0; *P* = 0.41; Supplementary Fig. 6) or between participants with lesions to each Yeo resting state functional network (*F*(6,432) = 0.5; *P* = 0.78; Supplementary Fig. 7). There was no difference in performance of the secondary outcome measure between participants with or without brain lesions (CVLT Immediate Recall Trial 1: *P* = 0.17).

### White matter integrity and attentional test performance

**Figure 3:**
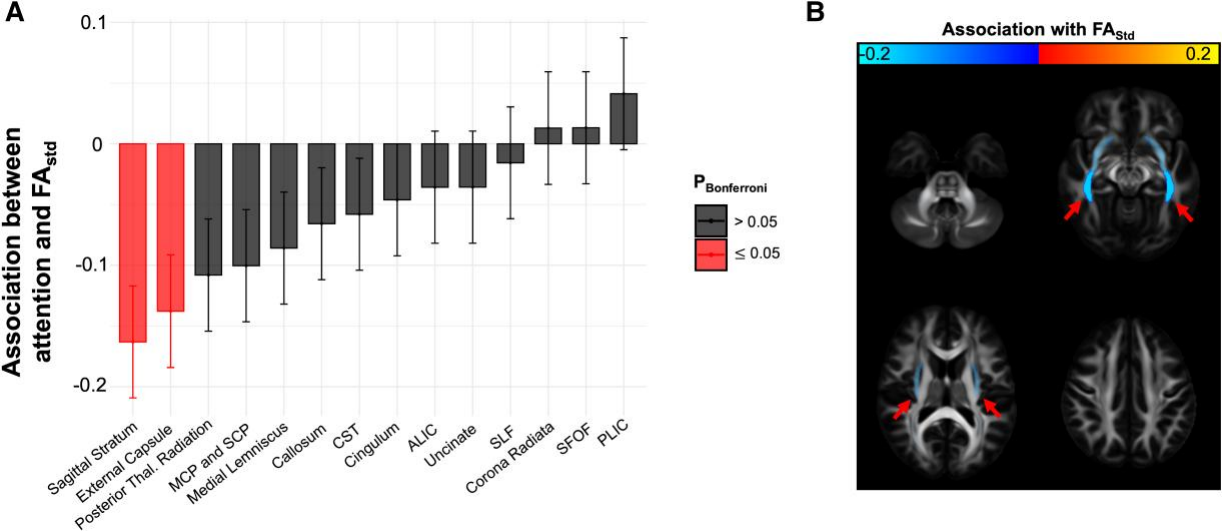
**Attentional test performance is associated with reduced white matter integrity in the sagittal stratum and external capsule.** (**A**) Bar plot showing the marginal association between Trails A *t*-score and each white matter ROI’s standardized FA (FA_std_; *N* = 182). The error bars indicate the standard error of the marginal association. The two bars on the left (red) correspond to a Bonferroni-adjusted *P*-value of <0.05 (corrected for 14 comparisons). (**B**) White matter regions are shown on an MNI T1 1 mm brain, with a colour corresponding to the strength of the marginal association (length of each bar in **A**). The intensity is scaled by the unadjusted *P*-value, such that a *P*-value of 0.05 is completely transparent and *P*-values smaller than 0.001 are completely opaque. Red arrows correspond to the sagittal stratum. ALIC, anterior limb of the internal capsule; CST, cortico-spinal tract; MCP, middle cerebellar peduncle; PLIC, posterior limb of the internal capsule; SCP, superior cerebellar peduncle; SFOF, superior fronto-occipital fasciculus; SLF, superior longitudinal fasciculus; Thal, thalamus.

There was a significant interaction between white matter regional integrity and Trails A performance (Trails A *t*-score × ROI: *F*(13,2330.1) = 2.6; *P* = 0.001; Table 2). The strongest *post hoc* marginal associations between Trails A *t*-score and regional FA were measured in the sagittal stratum and, to a lesser extent, the immediately adjacent external capsule (Fig. 3). Controlling for age, scanner, contrast-to-noise ratio and in-scanner movement, a 1 SD decrement in Trails A *t*-score performance (10-point increase) was associated with a −0.2 SD (−0.3, −0.1) reduction in sagittal stratum FA (*P*_Bonferroni_ = 0.0057) and a −0.1 (−0.2, −0.1) reduction in external capsule FA (*P*_Bonferroni_ = 0.042).

**Table 2:** Mixed ANOVA for attentional test performance and white matter integrity

	Degrees of freedom	Sum Sq	Mean Sq	*F*-value	*P*-value
Age	1	3.5	3.5	7.3	0.008
Scanner	2	19.9	9.9	20.5	<0.001
CNR	1	5.5	5.5	11.4	<0.001
Absolute motion	1	11.6	11.6	23.8	<0.001
Attentional test performance^a^	1	1.7	1.7	3.6	0.06
ROI	13	16.1	1.2	2.5	0.002
Attentional test performance × ROI	13	16.3	1.3	2.6	0.001

CNR, contrast-to-noise ratio; Mean Sq, mean of squared errors; ROI, region of interest; Sum Sq, sum of squared errors.

^a^Trails A *t*-score/10.

Splitting the non-midline regions by hemisphere, we found no significant interaction between Trails A *t*-score and hemisphere (*F*(1,6252.2) = 3.0; *P* = 0.08) or between Trails A *t*-score, region and hemisphere (*F*(17,6252.1) = 1.1; *P* = 0.3). Furthermore, the interaction between white matter region and Trails A performance, as well as the identification of the sagittal stratum as the most significant region, persisted after controlling for multiple markers of physical and motor functioning and after controlling for time between injury and assessment (Supplementary Table 3).

Using the secondary outcome measure, we also identified a significant interaction between CVLT Immediate Recall Trial 1 performance and regional white matter integrity (*F*(13,2340.9) = 2.6; *P* = 0.001; Supplementary Table 4). The strongest *post hoc* marginal associations between CVLT performance and white matter integrity were found in the corpus callosum [1 SD decrement in CVLT ∼ 0.2 (0.1, 0.3) SD change in FA *P*_Bonferroni_ = 0.014] and cingulum bundle [0.1 (0.0, 0.2); *P*_Bonferroni_ = 0.07; Supplementary Fig. 8], although the latter failed to meet strict Bonferroni significance criteria. The interaction between region and CVLT remained significant after additionally controlling for time since injury (*F*(13,2236.9) = 2.1; *P* = 0.01).

### Traumatic brain lesions versus white matter integrity

Finally, we tested whether encephalomalacic lesions and/or white matter integrity were independently associated with Trails A performance. White matter integrity in the sagittal stratum [1 SD decrease in FA ∼ 0.4 (0.1, 0.7) SD decrement in Trails A *t*-score; *P* = 0.008], but not encephalomalacic lesions [0.2 (−0.2, 0.7); *P* = 0.3], was independently associated with Trails A performance.

## Discussion

In this multicentre, cross-sectional cohort study of individuals with chronic TBI, we found that encephalomalacic brain lesions and diminished white matter integrity were each associated with worse performance on the Trails A test, a commonly used measure of visual attention and processing speed. We did not find that participants with worse Trails A performance had generally reduced white matter integrity, but rather reduced integrity within a specific region of temporal white matter known as the sagittal stratum^53^ and the immediately adjacent external capsule. Reduced white matter integrity within the sagittal stratum was independently associated with Trails A performance, even after controlling for the presence of encephalomalacic lesions, motor impairment and time since injury. In contrast, we did not find empirical support for the idea that traumatic lesions to specific grey matter regions or networks produce attentional impairment. Collectively, these observations indicate that the specific anatomical distribution of diminished white matter integrity after TBI may be pathophysiologically relevant to attentional performance.

While participants with encephalomalacic brain lesions (likely resulting from contusive injury) had slightly worse Trails A performance (not CVLT Trial1) than those without lesions, we did not identify a consistent relationship between lesion size and attentional performance. Indeed, participants with >40 cc lesions did not have measurable difference in Trails A performance compared with subjects with smaller than 4 cc lesions (Supplementary Fig. 4). Although this finding would seem to highlight the importance of injury to specific grey matter regions (rather than total burden of injury), we did not find variability in attentional performance based on which grey matter region was lesioned. Given that only 70 participants had encephalomalacic lesions, we were likely underpowered to rigorously test this hypothesis.

Prior studies have identified associations between multiple grey and white matter regions and performance on attentional testing.^15,22,54-57^ Our analytical approach differed from these previous studies by directly testing (and in the white matter, supporting) the hypothesis that injury to different brain regions produces differential effects on attention. While such a hypothesis may seem intuitively true (one would assume that diminished integrity of fibres within the posterior limb of the internal capsule, for example, would not affect attention), this has not yet been empirically demonstrated in a large and heterogeneous sample of patients with TBI.

Our analysis of the relationship between white matter integrity and Trails A performance identified the sagittal stratum and the external capsule as regions of peak association. The strongest association was in the sagittal stratum, a complex region located supero-laterally to the temporal horn of the lateral ventricle.^53^ This association was robust to adjustment for possible motor and non-motor confounding factors. There is debate about its boundaries, composition and function, but the sagittal stratum is believed to contain association fibres from the inferior longitudinal fasciculus and the inferior fronto-occipital fasciculus, which connect the frontal, parietal, temporal and occipital lobes, as well as projection fibres connecting these structures with the thalamus and brainstem.^53,58,59^ Fibres in the sagittal stratum are densely connected with the intralaminar thalamic nuclei,^60^ long postulated to play a role in sustaining arousal.^10^ The most common symptoms produced by invasive electrical stimulation of the sagittal stratum in humans are neglect and visual agnosias, deficits characterized by targeted loss of attention.^61^

In further support of this anatomic localization, the second strongest effect in this study was measured in the external capsule, which lies immediately anterior to the sagittal stratum. The external capsule sits between the putamen medially and the claustrum laterally and is known to carry cortico-cortical association fibres like the inferior fronto-occipital fasciculus,^62,63^ cortico-claustrum fibres^63^ and possibly also cholinergic projection fibres from the basal forebrain.^64^ The fronto-occipital fasciculus within this region may be one of the only direct connections between the frontal lobe and occipital (visual) areas in the human brain.^65^ Stimulation studies and anatomical dissections have suggested fascicles traversing the external capsule may play a role in reading, attention and visual processing.^66-69^ The third strongest association was in the posterior thalamic radiation, a bundle immediately dorsal to the sagittal stratum, important for relay of visual information, and previously found to be abnormal in tasks measuring visual working memory.^70^ Diminished integrity of fibres within this general white matter region (sagittal stratum, external capsule and posterior thalamic radiation) may therefore produce the greatest impairments in tasks requiring visual attention and may be causally relevant for impairments in processing speed and sustained attention that are common after TBI.

Only reduced white matter integrity, not the presence encephalomalacic lesions, was associated with worse performance on a secondary measure of attention, CVLT Immediate Recall Trial 1. This component of the CVLT relies on auditory (rather than visual) attention, in addition to language comprehension and working memory. We found that the association between CVLT performance and white matter integrity also varied by region. The peak associations were measured in the corpus callosum and cingulum bundle, regions not identified in the Trails A analysis.

The corpus callosum is commonly identified in brain–behaviour correlation studies as it is the largest white matter bundle in the brain, making it the most sensitive area to detect changes in white matter integrity.^71-73^ Impairments in callosal white matter integrity have previously been associated with broad impairments in cognition across multiple disease states.^72,74,75^ The second strongest white matter association with the CVLT Trial 1 was in the cingulum, analysed here as a single, averaged white matter structure. The parahippocampal cingulum has an established role in episodic memory, and the dorsal cingulum may play a role in various cognitive and mood-related processes.^76^ Sustained impairments in selective attention have been observed following anterior cingulotomy.^77^ The different white matter localization of Trails A and CVLT performance highlights the differing cognitive demands of these two attentional tasks. It reinforces the idea that a concept like ‘attention’ may in fact refer to multiple distinct cognitive systems with varying degrees of distributed processing.^78^

These preliminary findings provide anatomic guidance that may narrow the scope of therapeutic targets in future studies. Given that the sagittal stratum is a white matter region containing multiple crossing fibre bundles, a key area for future research will be to identify which of these bundles connect the cortical and subcortical structures required for sustaining attention. Future work should additionally assess whether disruptions to these white matter regions produce attentional impairment after other types of brain injury.

### Limitations

This work should be interpreted in the context of its limitations. First, we used bilaterally averaged ROIs. While averaging bilateral cortical and white matter regions reduces the number of statistical tests required, it also reduces the sensitivity to identify hemisphere-specific associations. Second, as previously discussed, given that only 40% of participants had focal cortical lesions, we likely had insufficient power to investigate the relevance of specific cortical regions and networks. Third, the dMRI-measured FA signal is susceptible to confounding by multiple non-physiological variables. While our analyses controlled for many of these known confounders, like head motion, signal quality and scanner type, it is possible that additional unmeasured variables produced residual confounding. For example, small registration errors are difficult to quantitate and could theoretically covary with attentional performance (e.g. more atrophy creates small degrees of ROI misalignment, leading to lower regional FA). While algorithmic harmonization^79^ can help mitigate some of these concerns, we lacked the matched control samples required to faithfully deploy such procedures. Fourth, the large fraction of enrolled participants with missing data could have created an analytic sample not entirely representative of the complete study population. Fifth, participants were mostly white and male, limiting our confidence in extrapolating the findings into other demographic groups. Sixth, the lack of longitudinal behavioural data in these analyses limits our understanding of the static versus progressive nature of observed impairments. Finally, there was variability in the time of post-injury assessment in this chronic TBI sample. It is possible that attentional impairment many years after a TBI is due to different factors than impairment seen in the early years.

## Conclusions

In this cross-sectional, multicentre cohort study, we found that brain lesions and focal reductions in white matter integrity are associated with attentional test performance in chronic TBI. Within the white matter, diminished integrity of white matter in the sagittal stratum showed the strongest association with attentional performance. Further investigations are needed to confirm this finding and determine which specific connections within the sagittal stratum are producing the impairment.

## Supplementary Material

fcae420_Supplementary_Data

## Data Availability

Data are available to investigators upon reasonable request.
