# Supplementary material for: Cortical lesions and focal white matter injury are associated with attentional performance in chronic traumatic brain injury
Source: Brain Commun. 2024 Nov 21;7(1):fcae420. doi: 10.1093/braincomms/fcae420 (PMC11806419; doi:10.1093/braincomms/fcae420)
Supplement: fcae420_Supplementary_Data [file fcae420_supplementary_data.pdf]

SUPPLEMENTARY MATERIALS

**Supplementary Table 1:** Diffusion MRI acquisition parameters			
	**Skyra**	**Achieva**	**Ingenia**
Diffusion directions	30	32	64
B_0 volumes	1	1	9
Voxel size	2mm isotropic	2mm isotropic	1.875x1.875x2.0mm
Repetition Time	7 seconds	7.85 seconds	3.6 seconds
Echo Time	0.089 seconds	0.07 seconds	0.084 seconds

**Supplementary Table 2:** Multivariate associations with whole white-matter fractional anisotropy (FA)		
**Variable**	**Multivariate Beta* [95% CI]**	**P value**
Scanner Type		
Skyra vs Achieva	-1.1 [-1.8, -0.4]	**0.002**
Ingenia vs Achieva	-1.0 [-1.4, -0.6]	**<0.001**
Absolute Motion	-0.89 [-1.3, -0.5]	**<0.001**
CNR**	1.1 [0.3, 1.9]	**0.007**
Outlier Slices	-0.09 [-0.4, 0.2]	0.6
Age	-0.01 [-0.02, -0.001]	**0.03**

*Coefficient reflects linear association with standardized mean whole white matter FA. A coefficient of 1 refers to a 1 standard deviation increase in FA.
**Contrast to Noise Ratio of the b0 Volume

**Supplementary Table 3:** Confounder Adjustment		
**Confound Variable**	**Trails A * ROI**	**Marginal Association Sag Stratum FA ~ Trails A**
Rand Physical Functioning T score	$F_{(13,2278.0)}$=2.6; p = 0.001	-0.17 [-0.26, -0.08]
Rand Physical Limitation T score	$F_{(13,2278.0)}$=2.6; p = 0.001	-0.16 [-0.25, -0.07]
UPDRS part 3 total T score	$F_{(13,2291.2)}$=2.6; p = 0.001	-0.13 [-0.23, -0.04]
Dominant hand grip strength T score	$F_{(13\ 2265.1)}$=2.4; p = 0.003	-0.14 [-0.24, -0.05]
Years since injury	($F_{(13,2226.1)}$ = 2.4; p=0.004)	-0.17 [-0.26, -0.07]

Abbreviations: FA fractional anisotropy; ROI region of interest; UPDRS Unified Parkinson's Disease Rating Scale.

**Supplementary Table 4:** Mixed ANOVA for attentional performance (CVLT Immediate Recall Standardized Score) and white matter integrity					
	Degrees of Freedom	Sum Sq	Mean Sq	F value	P value
Age	1	3.5	3.5	7.7	0.008
Scanner	2	19.6	9.8	20.2	< 0.001
CNR	1	5.9	5.9	12.3	0.001
Absolute Motion	1	8.4	8.4	17.4	< 0.001
CVLT	1	0.3	0.3	0.7	0.4
ROI	13	4.3	0.3	0.7	0.8
CVLT*ROI	13	16.4	1.3	2.6	0.001

Abbreviations CVLT: California Verbal Learning Test, CNR: contrast noise ratio, ROI: region of interest, Sum Sq: sum of squared errors, Mean Sq: mean of squared errors

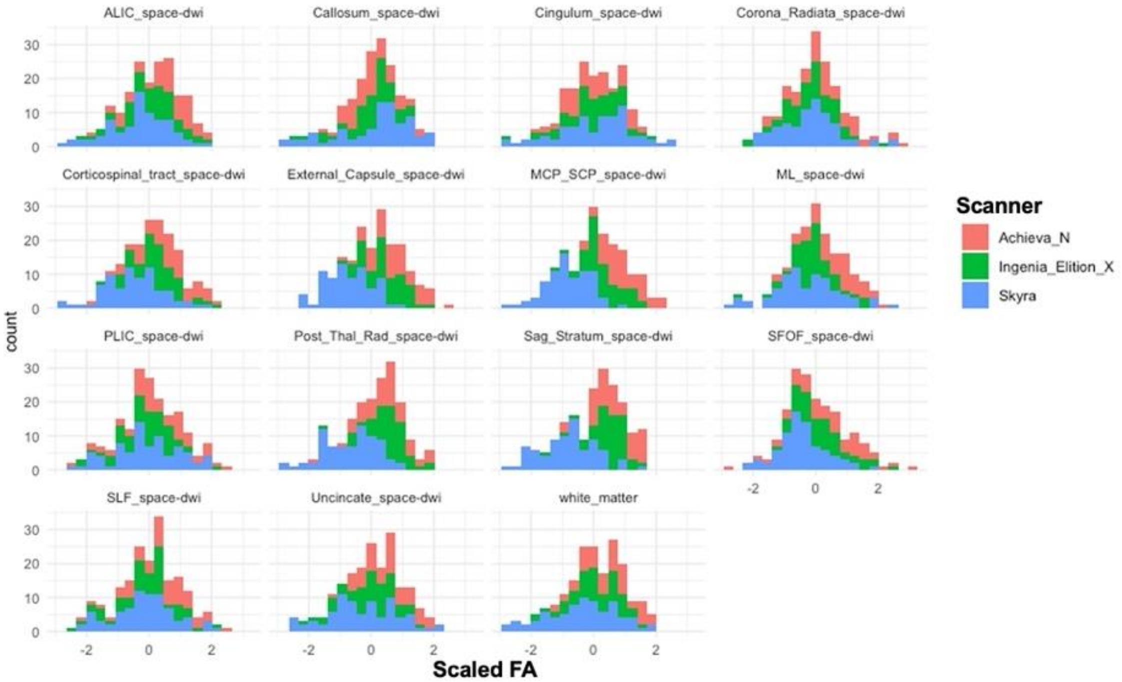

Supplementary Figure 1: Scaled FA by study scanner
The distribution of FA values, scaled to unit variance, within each white matter region of interest are shown. Color indicates the scanner on which the data were acquired.

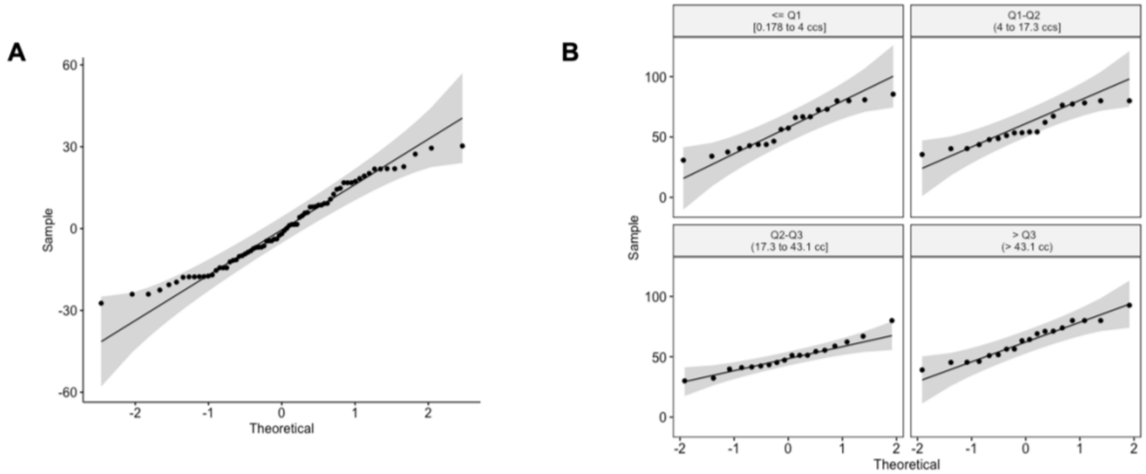

Supplementary Figure 2: Residual plots from analysis of association between lesion volume quartile and attentional performance
(A) Quartile-Quartile (QQ) plots of the distribution of Trails A t-scores in normally distributed (x axis) versus observed data (y axis), including only participants with encephalomalacic brain lesions. (B) QQ plots of the same data stratified by lesion volume quartile.

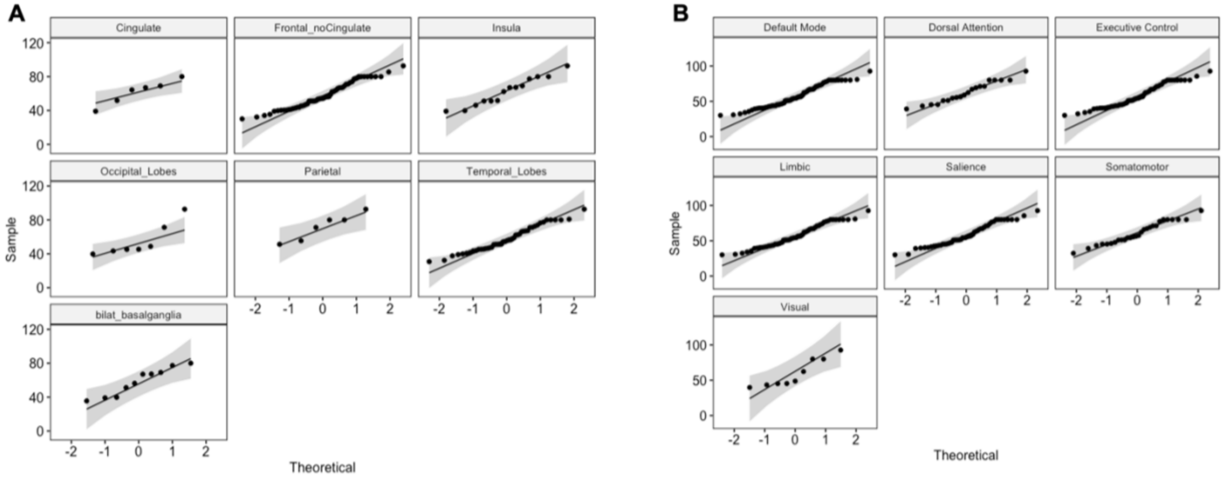

Supplementary Figure 3: Residual plots from analysis of association between lesion location and attentional performance
(A) Quartile-Quartile (QQ) plots of the distribution of Trails A T scores in normally distributed (x axis) versus observed data (y axis), including only participants with encephalomalacic brain lesions and stratified by the MNI atlas-based lesion location. (B) QQ Plots of the same data, stratified by Yeo-Network based lesion lesion.

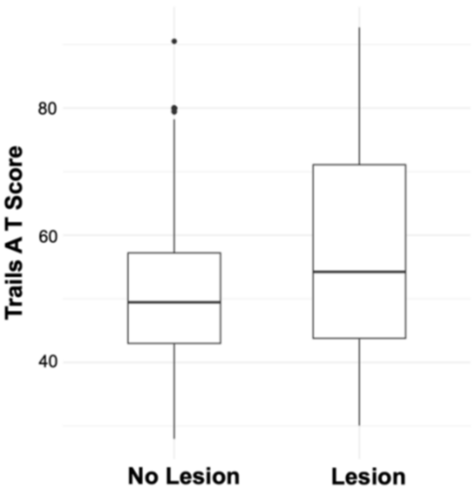

Supplementary Figure 4: Participants with encephalomalacic brain lesions had worse attentional performance.
Trails A t-scores in participants with or without brain lesions. Horizontal bar indicates median, box borders indicate 25th and 75th percentiles. Bar spans 1.5 IQR above and below and below the 25th and 75th percentiles. Observations outside that range are shown as individual points. Patients with lesions had higher (worse) Trails A t-scores (t-test: t=-2.1; p = 0.04).

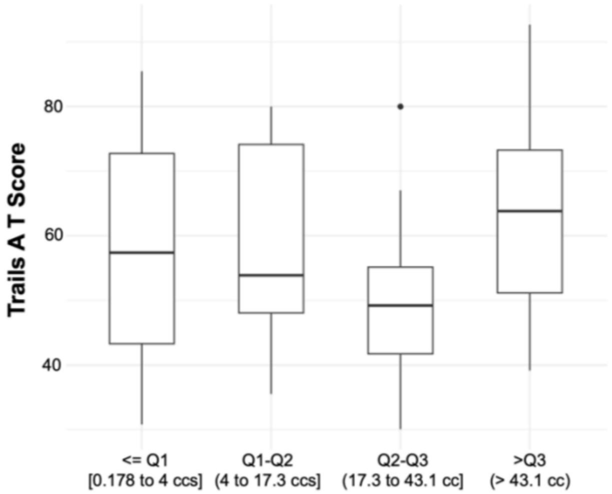

Supplementary Figure 5: Attentional performance does not differ by brain lesion volume quartile.
Trails A T scores in participants with brain lesions in each lesion volume quartile. The volume range comprised by the quartile (Q) is listed just beneath the x-tick label. A difference in the group means was tested using repeated measures ANOVA (N=73), with resulting $F_{3,561.6}$=2.4; p=0.07. Horizontal bar indicates median, box borders indicate 25th and 75th percentiles. Bar spans 1.5 IQR above and below and below the 25th and 75th percentiles. Observations outside that range are shown as individual points.

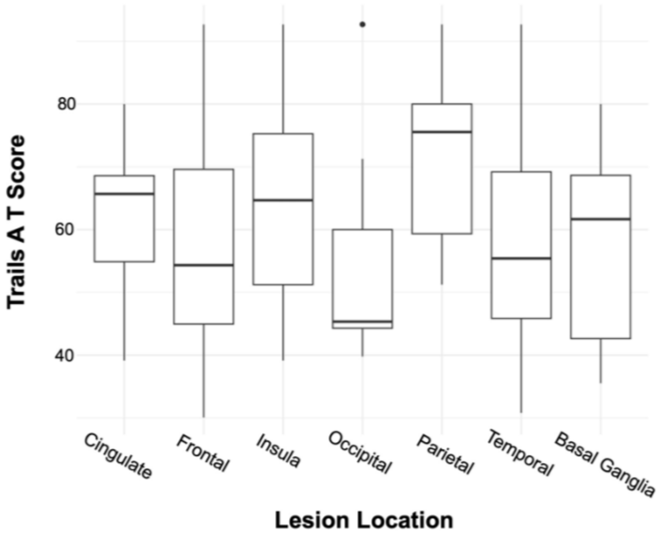

Supplementary Figure 6: Attentional performance does not differ among participants with lesions to different brain structures.
Trails A T scores in participants with brain lesions involving different Montreal Neurological Institute atlas cortical and subcortical regions. A difference in the group means was tested using repeated measures ANOVA, with a resulting p=0.41. Horizontal bar indicates median, box borders indicate 25th and 75th percentiles. Bar spans 1.5 IQR above and below and below the 25th and 75th percentiles. Observations outside that range are shown as individual points.

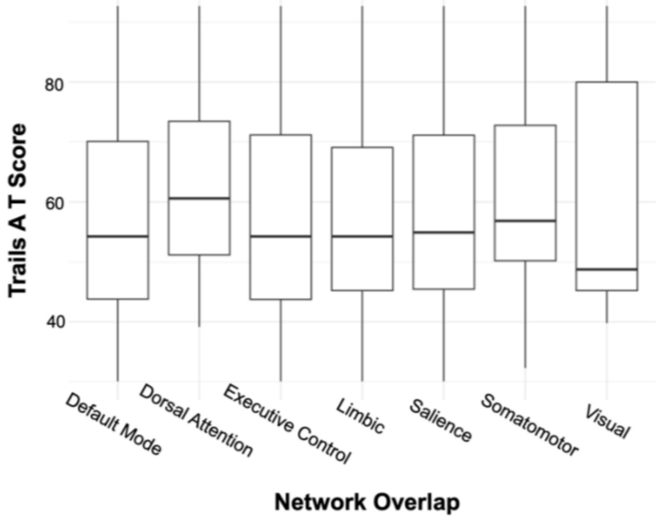

Supplementary Figure 7: Attentional performance does not differ among participants with lesions to different functional networks
Trails A T scores in participants with brain lesions that overlapped different Yeo cortical functional networks. A difference in the group means was tested using repeated measures ANOVA, with a resulting p=0.78. Horizontal bar indicates median, box borders indicate 25th and 75th percentiles. Bar spans 1.5 IQR above and below and below the 25th and 75th percentiles. Observations outside that range are shown as individual points.

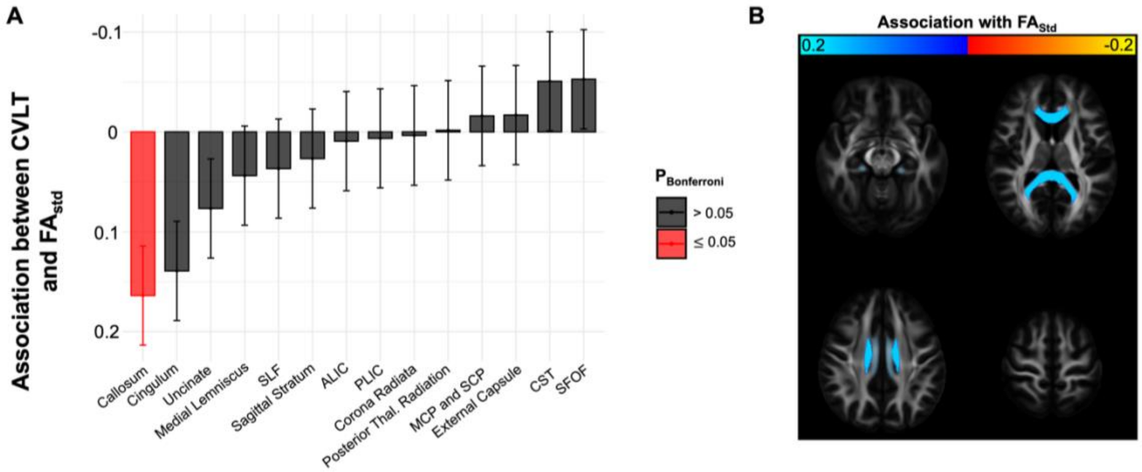

Supplementary Figure 8: CVLT Trial 1 performance is associated with reduced white matter integrity in the corpus callosum. (A) Bar plot showing the marginal association between CVLT Trial 1 standardized score and each white-matter region of interest's standardized fractional anisotropy (FAstd; N=182). The error bars indicate the standard error of the marginal association, and red bars indicate a Bonferroni-adjusted p value of < 0.05 (corrected for 14 comparisons). (B) White matter regions are shown on an MNI T1 1mm brain, with a color corresponding to the strength of the marginal association (length of each bar in [A]). The intensity is scaled by the unadjusted p value, such that a p value of 0.05 is completely transparent and p values smaller than 0.001 are completely opaque. Red arrows correspond to the sagittal stratum. Abbreviations: Thal: Thalamus, CST: Corticspinal Tract, MCP: Middle Cerebellar Peduncle, SCP: Superior Cerebellar Peduncle, ALIC: Anterior Limb of the Internal Capsule, SFOF: Superior Fronto-Occipital Fasciculus, SLF: Superior Longitudinal Fasciculus, PLIC: Posterior Limb of the Internal Capsule.
